# Effects of fertilization media on *in vitro* embryonic development in red-rumped agouti () using a heterologous system with guinea pig oocytes

**DOI:** 10.5935/1518-0557.20250180

**Published:** 2026

**Authors:** Lhara R. M. de Oliveira, Leonardo V. C. de Aquino, Luana G. P. Bezerra, Moacir F. de Oliveira, Alexandre R. Silva, Alexsandra F. Pereira

**Affiliations:** 1 Laboratory of Animal Biotechnology, Federal Rural University of Semi-Arid, Mossoró, RN, Brazil; 2 Laboratory of Animal Germplasm Conservation, Federal Rural University of Semi-Arid, Mossoró, RN, Brazil; 3 Laboratory of Applied Animal Morphophysiology, Federal Rural University of Semi-Arid, Mossoró, RN, Brazil

**Keywords:** wildlife, reproductive biology, gamete interaction, *in vitro* fertilization, epididymal sperm

## Abstract

**Objective:**

This study aimed to evaluate the effects of different heterologous *in vitro* fertilization (he-IVF) media on embryonic development in red-rumped agoutis (*Dasyprocta leporina*) using guinea pig oocytes as a model. Considering the species’ ecological and biotechnological relevance, optimizing IVF conditions is essential to improve assisted reproductive technologies for conservation and research.

**Methods:**

Epididymal spermatozoa from four red-rumped agoutis were selected and capacitated before co-incubation with *in vitro* matured guinea pig oocytes. Three IVF media were tested: human tubal fluid (HTF), Toyoda-Yokoyama-Hoshi (TYH), and Tyrode’s albumin lactate pyruvate (TALP). After six hours of fertilization, presumptive zygotes were cultured and evaluated on Days 2 and 5 for cleavage, morphology, assessments of oocyte-sperm interaction, and number of cells. In addition, reactive oxygen species (ROS) levels, and mitochondrial membrane potential assessments were performed to analyze oxidative stress during early development.

**Results:**

Among the tested media, TALP significantly improved cleavage rates (*p*<0.05) and resulted in a higher proportion of embryos with eight or more cells on both D2 and D5 of culture (*p*<0.05). Embryos derived from TALP also showed reduced oxidative stress, evidenced by lower ROS levels and decreased mitochondrial membrane potential compared to the other groups (*p*<0.05).

**Conclusions:**

TALP proved to be the most effective medium for supporting he-IVF using red-rumped agouti sperm, enhancing both developmental kinetics and cellular quality. These results contribute to the refinement of IVF protocols and represent an important step toward developing homologous embryo production systems in this neotropical species.

## INTRODUCTION

The red-rumped agouti (*Dasyprocta leporina* Linnaeus, 1758) is a wild rodent species with a stable population (Patton & Emmons, 2015) that holds significant ecological importance (Hadler *et al*., 2016) as a seed disperser and plays an economic role in the South American commerce (Hosken, 2001). Consequently, ongoing efforts are focused on refining assisted reproductive strategies, including *in vitro* fertilization (IVF), to enhance reproductive efficiency and advance our understanding of reproductive physiology (Mochida *et al*., 2014).

Following the initial phase of obtaining sperm samples from wild animals for IVF studies, the subsequent challenge lies in collecting a sufficient number of viable oocytes. This remains a significant obstacle due to the limited research available on optimizing *in vivo* oocyte retrieval techniques in wild species (Swanson, 2023). In this context, heterologous *in vitro* fertilization (he-IVF) offers a promising alternative for studying *in vitro* interactions, using oocytes from domestic species and sperm cells of wild animals (Cañón-Beltrán *et al*., 2023). The controlled environment of he-IVF allows us to assess valuable insights into cellular interactions during co-incubation and subsequent stages of embryonic development (Anifandis *et al*., 2014). Additionally, these findings could be applied to future homologous IVF in wild species.

Considering that the efficacy of he-IVF varies significantly among rodent species (Horie *et al*., 2015; Banafshi *et al*., 2021), it is essential to optimize key parameters, including sperm concentration, co-incubation duration, and, most importantly, the selection of an appropriate medium. These he-IVF media play a critical role in providing the necessary energy, protein, and ionic support to maintain gamete viability throughout co-incubation (Sciorio & Rinaudo, 2023), thereby fostering an environment that facilitates the essential metabolic processes required for successful fertilization and embryonic development (Swain *et al*., 2016). Additionally, these media help mitigate oxidative stress, which could be primarily induced by the sperm, preventing potential damage that could adversely affect subsequent stages of embryonic development (Castro *et al*., 2016).

Given their diverse compositions, he-IVF media can fulfill distinct functions across different species. For mouse embryo production, different concentrations of glucose, amino acids, sodium, and growth factors were found to reduce the percentage of blastocyst formation (Chronopoulou & Harper, 2015). This explains why variations in embryo outcomes are observed even among the most used media-Human Tubal Fluid (HTF), Toyoda-Yokoyama-Hoshi (TYH), and Tyrode’s Albumin Lactate Pyruvate (TALP). Suzuki & Fujinoki (2023) used TALP medium for IVF in *Mus musculus*, resulting in a 67% cleavage rate at the two-cell stage, but with no live births. In contrast, Mochida *et al*. (2014) used HTF medium, reporting 97% cleavage and successful births after embryo transfer. Similarly, González *et al*. (2024) found that the TYH medium resulted in lower fertilization rates and fewer blastocysts compared to HTF in mouse strands. However, Toyoda & Yokoyama (2016) demonstrated that increasing the volume of TYH medium during gamete co-incubation significantly improved fertilization success in the same species.

Therefore, this study aimed to elucidate the impact of IVF medium on the embryonic development of the red-rumped agouti using he-IVF model with guinea pig oocytes. This research seeks to contribute to the understanding of the reproductive physiology of this species and generate data that could support the future development of homologous IVF techniques for this wild rodent species.

## MATERIALS AND METHODS

The chemicals were procured from Sigma-Aldrich (St. Louis, MO, USA), unless otherwise specified.

### Bioethics

All experimental procedures were conducted in compliance with the guidelines established by the Animal Ethics Committee of the Federal Rural Semi-Arid (no. 20/2021) and Institute for Biodiversity Conservation (ICMBio, no. 76655-1).

### Experimental design

To determine the medium for he-IVF using red-rumped agouti spermatozoa and guinea pig oocytes, both gametes were assessed to ensure high quality. Epididymal spermatozoa were subjected to evaluation, including assessments of kinetic parameters, membrane functionality and viability, mitochondrial activity, DNA integrity, and morphological normality. The oocytes were confirmed to have reached maturation based on the expansion of *cumulus* cells and extrusion of the first polar body. Subsequently, gametes were co-incubated in microdroplets containing one of three distinct media formulations: Human Tubal Fluid (HTF), Toyoda-Yokoyama-Hoshi (TYH), and Tyrode’s Albumin Lactate Pyruvate (TALP) medium. Following a 6-hour incubation, presumptive zygotes were examined for morphological normality, interactions between oocytes and spermatozoa, kinetic parameters during early embryonic development up to the morula stage, and levels of oxidative stress throughout *in vitro* development (IVD).

A within-subjects experimental design was employed, in which samples of spermatozoa collected from red-rumped agoutis (n=4) and oocytes collected from guinea pig (n=12) were equally distributed among the IVF routines with four experimental groups per replicate (four replicates total).

### Animals and recovery procedures

The red-rumped agoutis and guinea pigs were kept at the Center of Multiplication of Wild Animals (CEMAS, UFERSA, Brazil). The animals were provided with *ad libitum* standardized commercial rabbit diet and fresh drinking water and maintained under a 12-hour natural photoperiod cycle.

For the recovery of spermatozoa, four sexually mature male red-rumped agouti specimens (one male/replicate) were captured and premedicated with a combination of ketamine (15 mg/kg; Ketalar, Pfizer, São Paulo, Brazil) and xylazine (1 mg/kg; Rompun, Bayer, São Paulo, Brazil). After a 15-minute interval, anesthesia was induced through intramuscular injection of sodium thiopental at a dosage of 50 mg/kg (Thiopentax; Cristalia, São Paulo, Brazil). Euthanasia was subsequently performed via intravenous administration of potassium chloride at a dosage of 1 mL/kg (Equiplex, Goiânia, Goiás, Brazil) (Castelo *et al*., 2015). For oocyte retrieval, twelve adult female guinea pigs (three females/replicate) were captured, anesthetized, and euthanized with the same protocol as the red-rumped agoutis.

### Sperm preparation for he-IVF

The testes-epididymis complex was retrieved immediately following euthanasia and immediately transported to the laboratory in a pre-warmed saline solution (37°C, 0.15 M NaCl). The cauda epididymis region was carefully dissected, and epididymal spermatozoa were extracted via retrograde flushing using 1.0 mL of saline solution (0.15 M NaCl) (Castelo *et al*., 2015). The collected sperm samples were maintained in a water bath at 37°C, while initial assessments of appearance, color, pH, vigor, and concentration were conducted.

The spermatozoa concentration was adjusted to 100×10^6^ sperm/mL using a minimum capacitation medium (MCM) composed of 105.8 mM NaCl, 25 mM NaHCO₃, 5.56 mM glucose, 21.6 mM sodium lactate, 25 mM HEPES, 0.25 mM sodium pyruvate, 10 µg/mL phenol red, and 1% antibiotic-antimycotic solution. A centrifugation protocol was employed to isolate the most viable gametes. A 1:1 mixture of spermatozoa and MCM was transferred to a 15 mL plastic tube and subjected to two centrifugation cycles (300× g for 3 min, room temperature). Following each centrifugation, the supernatant was carefully discarded, and the resulting pellet was resuspended for the he-IVF (Oliveira et al., 2025a).

Additionally, sperm capacitation was conducted simultaneously with the 6-hour he-IVF process. The capacitation agents employed included 4 mg/mL bovine serum albumin (BSA) and 2 mM calcium chloride (CaCl₂), both of which were incorporated into the he-IVF media within droplets overlaid with mineral oil (Oliveira et al., 2025b). The procedure was performed under controlled environmental conditions of 38.5°C and 6.5% CO_2_.

### Oocyte preparation for he-IVF

The ovaries were collected, stored in pre-warmed saline solution (0.15 M NaCl, 37°C), and immediately transported to the laboratory. All follicles were sliced under stereomicroscopic guidance to retrieve oocytes. Oocytes with one to four *cumulus* cell layers and homogeneous cytoplasm were selected for *in vitro* maturation (IVM) and matured in drops (100 µL) covered with mineral oil in a controlled atmosphere at 38.5°C and 6.5% CO_2_ for 24 h (Wang *et al*., 2019).

The oocyte maturation medium was composed of TCM199 supplemented with 2.2 g/L sodium bicarbonate, 25 mM HEPES, 0.2 mM sodium pyruvate, 100 µM cysteamine, 50 ng/mL epidermal growth factor (EGF), 10 µg/mL follicle-stimulating hormone associated with luteinizing hormone (FSH/LH; Pluset^®^, Hertape Calier, Juatuba, MG, Brazil), 10% fetal bovine serum (FBS), and 1% antibiotic-antimycotic solution (Wang *et al*., 2019). Following 24-hour maturation, matured oocytes were washed and grouped (10-20 oocytes per droplet) in preparation for he-IVF with the prepared spermatozoa.

### he-IVF and *in vitro* development (IVD)

The he-IVF droplets (50 µL) were prepared with three different IVF media: HTF, TYH, and TALP (Table 1). The droplets covered with mineral oil were balanced at 38.5°C in 6.5% CO_2_ for approximately 30 min before adding the prepared sperm with a final concentration of 1.0×10^6^ sperm/mL to 10-20 intact *cumulus*-enclosed matured oocytes (Cañón-Beltrán *et al*., 2021). Following a 6-hour co-incubation, structures were gently washed and pipetted to eliminate residual spermatozoa and *cumulus* cells.

**Table 1:** The three different IVF media compositions used for he-IVF.

Chemicals	HTF^1^	TYH^2^	TALP^3^
NaCl	101.6 mM	119.3 mM	114 mM
KCl	4.7 mM	4.7 mM	3.2 mM
NaH_2_PO_4_	-	-	0.35 mM
KH2PO4	0.37 mM	1.19 mM	-
Na lactate	21.6 mM	-	10 mM
Glucose	5.56 mM	2.78 mM	-
NaHCO_3_	25 mM	25 mM	25 mM
CaCl_2_	2 mM	2 mM	2 mM
MgSO_4_	0.20 mM	1.19 mM	0.50 mM
HEPES	25 mM	-	10 mM
BSA	4 mg/mL	4 mg/mL	4 mg/mL
Sodium pyruvate	0.33 mM	1 mM	0.11 mM
Phenol red	10 µg/mL	2 µg/mL	10 µg/mL
Antibiotic solution	1%	1%	1%

1 (Wigger *et al*., 2023).

2 (Toyoda & Yokoyama, 2016).

3 (Rath *et a*l., 1999). HEPES: 4-(2-hydroxyethyl)-1-piperazineethanesulfonic acid. BSA: Bovine Serum Albumin.

The structures were transferred for IVD medium drops (50 µL) at 38.5°C in 6.5% CO_2_ with Potassium Simplex Optimized Medium (KSOM: 95 mM NaCl, 2.5 mM KCl, 0.35 mM KH_2_PO_4_, 10 mM Na lactate, 0.2 mM glucose, 25 mM NaHCO_3_, 1.71 mM CaCl_2_, 0.20 mM MgSO_4_, 21 mM HEPES, 0.01 mM phenol red, 0.2 mM sodium pyruvate, 0.01 mM EDTA, 1 mM L-glutamine, 15 mM BSA, 1% antibiotic-antimycotic solution, 1% essential amino acid solution, 0.5% non-essential amino acid solution, and 10% FBS) (Lawitts & Biggers, 1993). After 48 h of culture (D2), 50% of the KSOM medium was changed. The total culture time was 120 h (D5) to evaluate development kinetics (Praxedes *et al*., 2023).

### Sperm, oocytes and zygotes evaluations

All gametes used for the he-IVF were systematically sampled and assessed to confirm their viability and suitability for the procedure. Following fertilization, the resulting structures were analyzed to identify the most effective experimental group. Each IVF routine was one replicate (4 total), with four experimental groups per replicate.

#### Sperm evaluation

The hypoosmotic swelling test (HOST) was performed using a solution consisting of distilled water (0 mOsm/L) with a sodium citrate and fructose solution (50 mOsm/L). Aliquots of 5 *µL* containing epididymal spermatozoa were combined with 45 *µL* of the hypoosmotic solution and incubated in a dry bath for 40 min at 37°C. The samples were then assessed under a phase-contrast light microscope at 400× magnification, with 100 cells analyzed per group/per replicate. Spermatozoa displaying a swollen and coiled tail were identified as possessing a functionally intact membrane (Dantas *et al*., 2022a).

A 10 µL aliquot of spermatozoa was incubated with 40 µg/mL Hoechst 33342 (Molecular Probes, Eugene, OR, USA) at 37°C for 5 min, followed by incubation with 0.5 mg/mL propidium iodide (Thermo Fisher Scientific, Whaltam, MA, USA) and 500 nM CMXRos (Mito Tracker Red^®^, F-7512, Molecular Probes, Eugene, OR, US) for 8 min. One hundred cells were evaluated in each experimental group (per replicate) using fluorescence microscopy (Olympus BX51TF, Tokyo, Japan) at 400× magnification. Spermatozoa exhibiting fluorescence with blue-stained head (350 nm) and a red-glowing midpiece (570 nm) were classified as possessing an intact plasma membrane and normal mitochondrial function (Santos *et al*., 2023).

The epididymal spermatozoa were evaluated using a computer-assisted sperm analysis (CASA) system (IVOS 7.4G; Hamilton-Thorne Research, MA, USA) with parameters previously established for red-rumped agouti. The settings included a temperature of 37°C, a straightness threshold of 30%, a minimum contrast of 45, a low-velocity average pathway (VAP) cutoff of 10 *µm*/s, and a medium VAP cutoff of 30 *µm*/s. Five independent and nonconsecutive microscopic fields were systematically examined. The following kinetic parameters were assessed: total motility (TM, %), progressive motility (PM, %), average path velocity (VAP, *µm*/s), straight-line velocity (VSL, *µm*/s), curvilinear velocity (VCL, *µm*/s), amplitude of lateral head displacement (ALH, *µm*), beat cross frequency (BCF, Hz), straightness (STR, %), and linearity (LIN, %). The sperm population was further categorized into four distinct groups: rapid, medium, slow, and static (%) (Castelo *et al*., 2015).

For DNA damage, sperm samples were prepared as smears and allowed to air-dry. The slides were subsequently fixed in Carnoy’s solution for 3 h, rinsed, and dried again at room temperature. They were then incubated for 25 min in a buffer solution consisting of 15 mM Na₂HPO₄ and 80 mM citric acid (pH 2.5) at 75°C. The smears were stained with acridine orange (0.2 mg/mL) for 10 s, rinsed with distilled water, and covered with coverslip. A total of 100 cells (per group/per replicate) were examined using fluorescence microscopy (480 nm, 400×; Olympus BX51TF, Tokyo, Japan). Spermatozoa with normal (double-stranded) DNA exhibited a green, fluorescent emission, while those with denatured or single-stranded DNA displayed yellow, orange, or red fluorescence, indicating progressively higher levels of DNA damage (Tomov *et al*., 2020).

For morphological assessment, a 10 µL aliquot of the sperm samples was fixed and stained using a formaldehyde-Bengal rose solution (Cromato^®^). The samples were then examined under a light microscope at 1000× magnification, with 100 cells evaluated (per group/per replicate). The spermatozoa were categorized into two groups: normal morphology and abnormal morphology. Abnormalities were further classified as defects in the head, midpiece, or tail regions (Silva *et al*., 2011).

#### Oocyte evaluation

Following 24-hour maturation, *cumulus*-oocyte complexes (COCs) were examined under a stereomicroscope to evaluate the extent of *cumulus* cell expansion. Structures demonstrating pronounced expansion and mucification were classified as mature. The presence or absence of the first polar body (1PB) was documented after removing excess *cumulus* cells through pipetting post-IVF. Oocytes exhibiting clear evidence of first polar body extrusion were identified as mature (Wang *et al*., 2019).

#### Zygotes evaluation

After a 6-hour IVF, each presumptive zygote was examined using an inverted microscope (Leipzig IMx 400, PhoenixOptics, Germany). The zygotes were carefully rotated to allow a clear assessment of their morphological features. Those displaying less than 20% of cytoplasmatic fragmentation, normal size, homogeneous cytoplasm and unruptured membrane were defined as normal (Hesters *et al*., 2008).

The cleaved heterologous zygotes were cultured for 120 h (up to Day 5) to assess developmental kinetics using an inverted microscope (Leipzig IMx 400, PhoenixOptics, Germany). Cell cleavage was evaluated and categorized into three groups: two cells, three to seven cells, or eight or more cells. Additionally, the percentage of morulae formation was quantified on Day 5, along with the ratio of morulae to total cleaved structures (Praxedes *et al*., 2023).

The non-cleaved cells were analyzed for oocyte-sperm interaction by staining with Hoechst 33342 (10 µg/mL, 30 min) and examined under a fluorescence microscope (350 nm). The number of spermatozoa attached to the zona pellucida, and the quantity associated with each oocyte were quantified, providing evidence of both monospermy and polyspermy (Santos *et al*., 2023).

To assess the oxidative stress response, reactive oxygen species (ROS) and mitochondrial membrane potential (∆Ψm) were quantified using 10 µM 2’,7’-dichlorodihydrofluorescein diacetate (H_2_DCFDA; 490 nm; Invitrogen, Carlsbad, CA, USA) and 500 nM MitoTracker Red^®^ (570 nm; Molecular Probes, Eugene, OR, USA), respectively. Both presumptive zygotes and morulae were incubated in the dark with the respective probes for 30 min at 38.5°C under 6.5% CO₂. Subsequently, the samples were placed on glass slides in microdroplets, and images were captured using a fluorescence microscope (Olympus BX51TF, Tokyo, Japan). Fluorescence intensity was quantified using ImageJ software (National Institutes of Health, Bethesda, Maryland, USA). The control group served as the calibrator, and the fluorescence values of each treatment were normalized to the mean of the calibrator to generate relative expression levels, expressed in arbitrary fluorescence units (AFU) (Santos *et al*., 2023).

### Statistical analysis

All data were expressed as mean±standard error (one male/one replicate) and analyzed using the GraphPad software (GraphPad Software Inc., La Jolla, CA, USA). All results were verified for normality using the Shapiro-Wilk test and for homoscedasticity using Levene’s test. Sperm interactions with oocytes and zygotes’ oxidative stress evaluations did not show a normal distribution; therefore, they were arcsine transformed and analyzed by ANOVA, followed by the Tukey test. All other data were compared with a chi-squared test. Significance was set at *p*<0.05.

## RESULTS

All flushed epididymal sperm samples exhibited a whitish color, with an average pH of 7.0 and a vigor score of 4.0 on a 0-5 scale. The mean concentration following selection was 53.0×10^6^ spermatozoa/mL. Furthermore, 24 ovaries were recovered, yielding 401 viable oocytes, which corresponds to an average of 33.4±7.5 viable oocytes per female.

### Sperm and oocyte preparation for he-IVF

**Figure 1:**
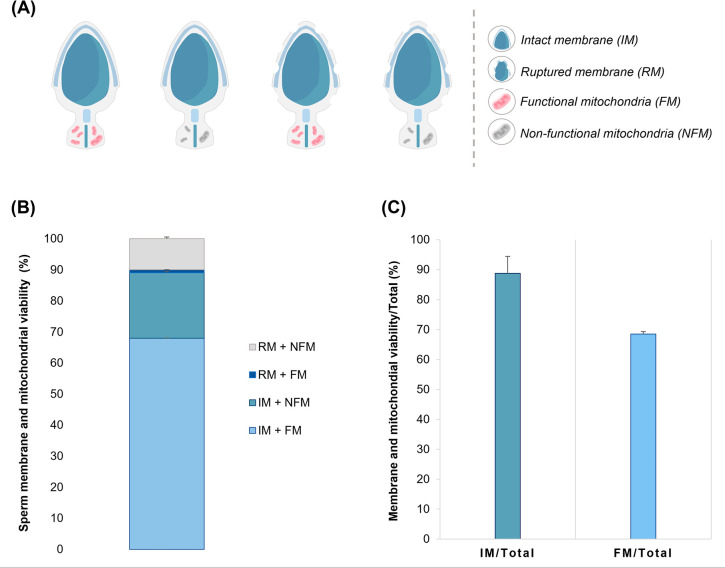
Sperm membrane viability and mitochondrial activity in red-rumped agoutis. (A) Representative illustration showing the parameters analyzed for sperm membrane viability and mitochondrial activity in red-rumped agouti. (B) **The rate of intact or ruptured red-rumped agouti sperm membrane and mitochondrial activity labeled with H33342, propidium iodide, and MitoTracker Red fluorescent probes.** (C) The percentage of sperm cells with intact membranes and normal mitochondrial activity relative to the total number of counted cells.

The CASA analysis demonstrated that the sperm used in he-IVF exhibited optimal and elevated rates across all evaluated parameters (Table 2), including total motility exceeding 96.0%, progressive motility surpassing 63.0%, and a substantial proportion of the sperm population classified as rapid (81.0%). The epididymal spermatozoa from red-rumped agouti demonstrated a high rate of membrane functionality (91.0%) by the HOS test following the selection process. The percentages of membrane integrity and mitochondrial activity (Figure 1A) were also notably high, with 68.0%±1.0 of the samples exhibiting both an intact membrane and functional mitochondria (Figure 1B). Lower proportions were observed for spermatozoa displaying loss of mitochondrial function (20.8%±4.7), rupture of the plasma membrane (0.5%±0.2), and both loss of mitochondrial function and membrane rupture (10.7%±5.4). These findings indicate that 88.8%±5.6 of the spermatozoa used in he-IVF presented an intact plasma membrane, while 68.5%±0.8 exhibited functional mitochondrial activity (Figure 1C).

**Table 2:** Computer-aided sperm analysis of red-rumped agouti epididymal sperm used for he-IVF.

CASA parameters	(mean±SE)
Total motility (%)	96.3±1.8
Progressive motility (%)	63.0±5.2
VAP (µm/s)	117.6±16.8
VSL (µm/s)	98.4±14.6
VCL (µm/s)	155.5±11.5
ALH (µm/s)	5.7±0.2
BCF (Hz)	29.5±2.8
STR (%)	77.8±1.6
LIN (%)	58.3±5.1
Rapid (%)	81.0±5.8
Medium (%)	15.5±4.5
Slow (%)	0.0±0.0
Static (%)	3.5±1.8

SE: standard error. VAP: velocity average pathway; VSL: velocity straight line; VCL: curvilinear velocity; ALH: amplitude of lateral head; BCF: beat cross frequency; STR: straightness; LIN: linearity. Sperm population: rapid, medium, slow or static.

No significant DNA damage was detected in the samples analyzed. Specifically, 87.5%±2.5 of the sperm exhibited intact DNA, while 5.0%±1.4 showed low DNA damage, 4.8%±0.8 exhibited moderate DNA damage, and only 2.8%±1.3 displayed high DNA damage. These findings were consistent with the sperm morphology parameters (Table 3), where more than 88.0% of the spermatozoa displayed normal morphology. The abnormalities observed were uniformly distributed across the three regions examined: head, middle piece, and tail.

**Table 3:** Sperm morphology analysis from red-rumped agouti epididymal sperm samples for he-IVF.

Sperm morphology Normal Abnormal	(%±SE) 88.8±2.9 11.2±2.9
**Abnormal morphology** Head defects Middle piece defects Tail defects	**(%±SE)** 2.5±0.6 5.3±3.3 3.4±0.2

SE: standard error.

**Figure 2:**
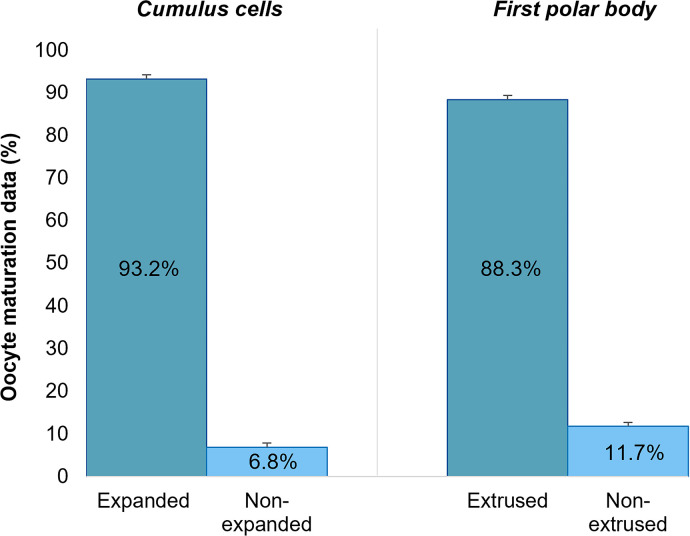
Efficiency of oocyte *in vitro* maturation (IVM) from guinea pigs for he-IVF analyzed by *cumulus* cell expansion and extrusion of the first polar body (1PB).

Concerning the viability of guinea pig oocytes (Figure 2), more than 93.0% exhibited substantial *cumulus* cell expansion following 24 h of maturation. Additionally, 88.3% of the oocytes exhibited 1PB extrusion, indicating nuclear maturity and suitability for he-IVF.

### he-IVF and *in vitro* development

**Figure 3:**
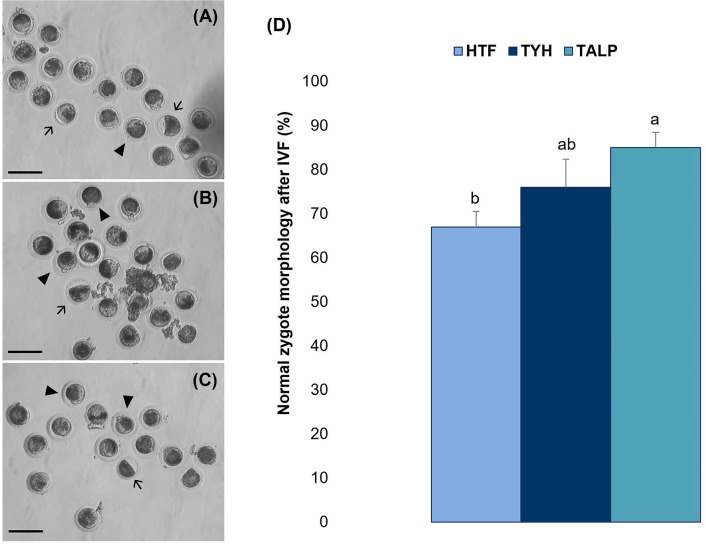
Impact of three distinct IVF media (HTF vs. TYH vs. TALP) on the morphological integrity of presumptive zygotes following a 6-h he-IVF protocol. (A) Representative images of presumptive zygotes fertilized in HTF, (B) TYH, and (C) TALP media. (D) Percentage of normal morphology zygotes after he-IVF. Arrows indicate zygotes with abnormal morphology; arrowheads represent zygotes exhibiting typical or desired morphological characteristics. Different letters (^a,b^) indicate a statistical difference (*p*<0.05). Scale Bar = 50 µm. Magnification: 20 ×.

The different he-IVF media significantly influenced the morphology of presumptive zygotes (Figure 3). The TALP group (85.0%±3.5) exhibited a higher rate of normal morphology compared to the HTF group (67.0%±3.5) (*p*<0.05). The TYH group (76.0%±6.4) displayed results comparable to those of both the TALP and HTF groups (*p*<0.05).

Comparable results were observed in embryo development kinetics (Table 4). On Day 2, the TALP group exhibited that 48.6% of the samples had cleaved after IVF, surpassing all other groups (*p*<0.05). Among the cleaved structures, the TALP group also demonstrated the highest percentage of embryos, with more than eight cells (*p*<0.05). On Day 5, the rate of cleaved structures was even higher in the TALP group (*p*<0.05), with 71.2% of cleavage observed compared to 29-38% in the HTF and TYH groups. Similarly, the TALP group predominantly exhibited cleavage with more than eight cells, outpacing the other treatments (*p*<0.05). Finally, no differences were observed between the groups regarding the percentage of morulae on Day 5 (*p* 0.05), with the total percentage ranging from 9.2% to 14.4%, and the total/cleavage percentage varying between 20.2% and 30.8%.

**Table 4:** He-IVF efficiency using three different IVF media with prepared red-rumped agouti sperm and matured guinea pig oocytes.

Groups	HTF	TYH	TALP
***Cleavage rate (D2)***			
Total cleavage (%)	25.3±4.7 (22/87) ^b^	19.4±1.3 (21/108) ^b^	48.6±2.7 (54/111) ^a^
2 cells (%)	16.1±3.5 (14/87) ^a^	10.2±3.8 (11/108) ^a^	18.0±1.0 (20/111) ^a^
3-7 cells (%)	9.2±2.6 (8/87) ^ab^	8.3±2.5 (9/108) ^b^	18.0±2.1 (20/111) ^a^
≥ 8 cells (%)	0.0±0.0 (0/87)^b^	0.9±0.6 (1/108) ^b^	12.6±1.2 (14/111) ^a^
***Cleavage rate (D5)***			
Total cleavage (%)	29.9±3.6 (26/87) ^b^	38.0±8.8 (41/108) ^b^	71.2±4.8 (79/111) ^a^
2 cells (%)	6.9±5.0 (6/87) ^a^	12.0±8.1 (13/108) ^a^	13.5±5.9 (15/111) ^a^
3-7 cells (%)	10.3±6.0 (9/87) ^b^	13.9±5.5 (15/108) ^ab^	23.4±2.1 (26/111) ^a^
≥ 8 cells (%)	12.6±4.7 (11/87) ^b^	12.0±3.4 (13/108) ^b^	31.5±2.5 (35/111) ^a^
***Morulae (D5)***			
Total (%)	9.2±3.2 (8/87) ^a^	9.3±3.3 (10/108) ^a^	14.4±1.8 (16/111) ^a^
Total/cleaved (%)	30.8±10.2 (8/26) ^a^	24.4±3.8 (10/41) ^a^	20.2±3.1 (16/79) ^a^

Mean±standard error.

a,b Values with different subscript letters are significantly different between treatments (*p*<0.05). HTF: Human Tubal Fluid medium. TYH: Toyoda-Yokoyama-Hoshi medium. TALP: Tyrode's Albumin Lactate Pyruvate medium. D2: Day two of *in vitro* culture. D5: Day five of *in vitro* culture.

**Figure 4:**
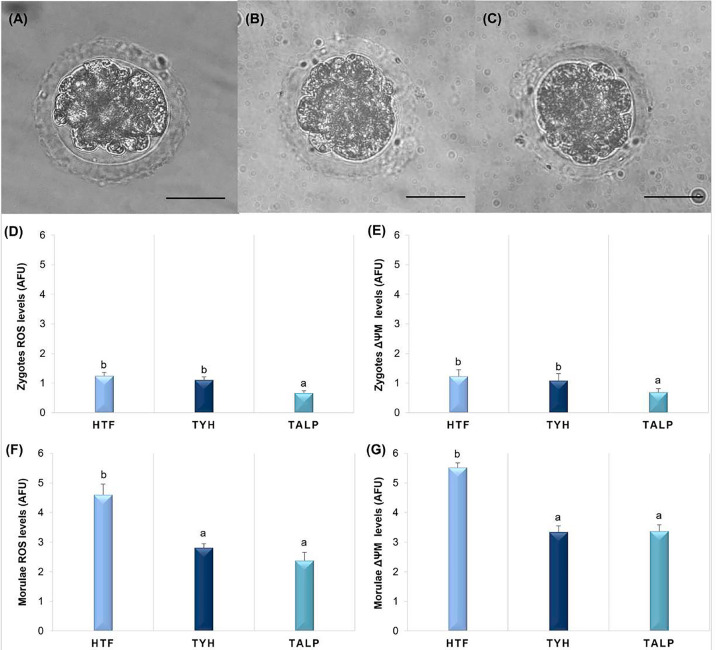
**Effect of different he-IVF media on presumptive zygotes and morulae ROS production and mitochondrial membrane potential (∆Ψm).** (A) **Representative image of morulae on D5 of culture after being fertilized in HTF,** (B) **TYH, and** (C) **TALP media.** (D) **ROS levels in arbitrary fluorescence units (AFU) from labeled zygotes with the H_2_DCFDA fluorescent probe.** (E) **∆Ψm levels from labeled zygotes with MitoTracker Red fluorescent probe.** (F) **ROS levels from labeled morulae.** (G) **∆Ψm levels from labeled morulae. Different letters (^a,b^) indicate a statistically significant difference between treatments (*p*<0.05). Scale Bar = 20 µm. Magnification: 40 ×.**

No differences (*p*>0.05) were observed in sperm-oocyte interaction following he-IVF (Table 5), with monospermy rates ranging from 93.1% to 98.2%. However, the evaluation of oxidative stress (Figure 4) revealed that zygotes fertilized with TALP media exhibited lower ROS production (0.66±0.08 AFU) compared to those fertilized with HTF (1.24±0.12 AFU) and TYH (1.10±0.11 AFU) (Figure 4D, *p*<0.05). Furthermore, the ∆Ψm was significantly lower in zygotes fertilized with TALP (0.69±0.12 AFU) than in those fertilized with HTF (1.23±0.22 AFU) and TYH (1.08±0.24 AFU) (Figure 4E, *p*<0.05). The morulae analysis indicated that both TYH (2.81±0.14 AFU) and TALP (2.37±0.29 AFU) media resulted in reduced ROS production compared to HTF (4.60±0.36 AFU) (Figure 4F, *p*<0.05). Similarly, ∆Ψm levels in morulae were lower in the TYH (3.34±0.21 AFU) and TALP (3.37±0.22 AFU) groups compared to HTF (5.52±0.16 AFU) (Figure 4G, *p*<0.05).

**Table 5:** Sperm-oocyte interaction during he-IVF between red-rumped agouti and guinea pig.

Groups	HTF	TYH	TALP
**Sperm/oocyte** **(mean±SE)**	1.1±0.1	1.0±0.0	1.1±0.1
**Sperm/zona pellucida** **(mean±SE)**	46.0±14.2	48.0±12.1	50.8±15.0
**Monospermy (%)**	93.1±4.7	98.2±1.8	93.8±6.3
**Polyspermy (%)**	6.9±4.7	1.8±1.8	6.3±6.3

Mean±standard error. *P* > 0.05. HTF: Human Tubal Fluid medium. TYH: Toyoda-Yokoyama-Hoshi medium. TALP: Tyrode's Albumin Lactate Pyruvate medium.

## DISCUSSION

The findings of this study substantiate that TALP serves as the most effective medium for supporting the entire he-IVF process using red-rumped agouti sperm and guinea pig oocytes. This medium effectively mitigated oxidative stress, as indicated by reduced ROS levels and lower ∆Ψm production in zygotes, thereby minimizing morphological abnormalities in these structures. Moreover, the use of TALP during he-IVF significantly promoted high cellular cleavage during IVD on Days 2 and 5, supporting the progression of cleaved zygotes to the full morula stage.

Successful IVF requires the use of viable and functional gametes. The high functionality of the sperm membrane is particularly significant, as this membrane plays a crucial role in sperm capacitation, acrosome reaction, sperm-egg fusion, and regulating fluid influx into the cell, thereby maintaining osmotic balance (Neamah, 2022). Our findings, which show higher rates of functionality, align with those of Dantas *et al*. (2022a, 2022b, 2024), who reported membrane functionality rates of approximately 80% when conducting the hypo-osmotic swelling test on cauda epididymal sperm from red-rumped agoutis.

The minimal loss of mitochondrial function following selection and capacitation was also a significant factor in maintaining the viability of red-rumped agouti spermatozoa. Mitochondria are essential for sperm motility, glycolysis, and oxidative phosphorylation, and serve as the primary source of pro-oxidative agents. It is hypothesized that dysfunction of this organelle plays a critical role in oxidative imbalance, which can compromise sperm function (Barbagallo *et al*., 2020). Dantas *et al*. (2022a, 2022b, 2024) reported similarly high rates of normal mitochondrial activity in red-rumped agouti epididymal sperm, with approximately 80%, aligning closely with our findings.

Among all parameters, sperm motility is recognized as a highly reliable predictor of male fertility potential (Dcunha *et al*., 2022). Our findings demonstrate a total motility (TM) exceeding 96%, a progressive motility (PM) of 63%, and more than 80% of the sperm population was classified as rapid. These results represent an improvement in the red-rumped agouti, surpassing the findings of previous studies. For instance, Castelo *et al*. (2015) observed only 90% motility with epididymal sperm, whereas Dantas *et al*. (2022a) reported a TM of 79.8% and PM of 13.7%. Additionally, Castelo *et al*. (2023) found a TM of just 24.8%. This enhancement can be attributed to the optimization of novel protocols for sperm selection and capacitation developed by our team and implemented during this study.

In recent decades, sperm DNA integrity has become one of the most widely discussed biomarkers in basic andrology, driven by the growing need for a more comprehensive understanding of sperm physiology. DNA damage is known to negatively impact fertilization, embryo development, implantation, and pregnancy outcomes (Zhu *et al*., 2022). Therefore, the high levels of intact DNA observed following recovery in this study confirm the viability of these samples for he-IVF. A similar conclusion can be drawn from the elevated rates of normal sperm morphology, underscoring the potential positive implications of preserving these morphological characteristics, which may be crucial for egg recognition, enhanced motility, and improved fertilization potential of these samples (Pelzman & Sandlow, 2024).

Ensuring proper oocyte maturation is a crucial factor in optimizing IVF protocols and *in vitro* development outcomes, as it enables the necessary cytoplasmic and nuclear modifications required for successful sperm interaction during fertilization (Fair & Lonergan, 2023). In our study, we achieved promising maturation rates exceeding 88.0%, aligning with previously reported values for this species. For instance, Wang *et al*. (2019) observed a metaphase II (MII) rate of over 61.0% after a 24-hour *in vitro* maturation period for guinea pig oocytes.

Furthermore, our findings suggest a correlation between successful oocyte maturation and improved embryonic development rates. Even under less efficient IVF conditions, we observed 19.0% cleavage and 9.0% morula formation, which remain within acceptable standards for wild species IVF (Ferraz *et al*., 2020). Similar associations between oocyte maturation and subsequent embryonic development have been reported by Praxedes *et al*. (2023). Their study on red-rumped agouti oocytes demonstrated that the IVM medium yielding the highest maturation rate (10 ng/mL epidermal growth factor with 10 µg/mL FSH, 52.1% MII) also resulted in a superior cleavage index on Day 2 (43.2%) following oocyte chemical activation. In contrast, oocytes that exhibited inadequate maturation (100 ng/mL epidermal growth factor with 10 µg/mL FSH, 37.5% MII) displayed compromised developmental potential, with only 15.0% cleavage by Day 2.

Upon comparing the three different media following the 6-hour IVF, TALP was identified as the most suitable for this species, as it yielded a higher number of normally formed zygotes and demonstrated superior *in vitro* development. While the identification of embryos with the highest implantation potential remains an unresolved challenge in reproductive science, numerous approaches have been proposed in recent years to evaluate embryo viability (Stigliani *et al*., 2021). In our study, we employed a commonly used method of selection based on the analysis of morphological features, including size, shape, and degree of fragmentation. Our findings revealed that the group exhibiting the most normal morphological structures also showed the highest cleavage rate, providing evidence for the efficacy of the method applied in this research.

Due to the limited availability of oocytes from wild species, he-IVF using oocytes from phylogenetically related species, such as the guinea pig, is a valuable strategy for evaluating sperm parameters in the red-rumped agouti. Regardless of the IVF media used, we observed satisfactory oocyte-sperm interaction, with a low incidence of polyspermy (~5%). This finding is particularly relevant, as polyspermy rates in IVF systems can exceed 10%, rendering the resulting embryos unsuitable for transplantation and compromising their subsequent development (Sun & Zhu, 2023).

By Day 2 of embryo development, the TALP-derived structures exhibited the highest number of ≥8 cell cleavage stages, which is significant, as slower cleavage rates are generally considered to affect implantation potential negatively (Hesters *et al*., 2008). Studies have shown that the timing of the first cleavage of the zygote can serve as an important criterion for selecting embryos with the highest implantation potential (Lechniak *et al*., 2008). Furthermore, the transfer of embryos derived from early cleaved zygotes has been associated with higher pregnancy and implantation rates compared to the transfer of embryos from non-early cleaved zygotes (Almagor *et al*., 2015).

Following IVF, the three media appeared to exert distinct effects on *in vitro* embryonic development. The structures fertilized in HTF exhibited slower cleavage, with no structures surpassing eight cells by Day 2. It took a total of 120 hours for the structures to further develop; however, by Day 2, all cleaved structures appeared to have reached the morula stage, resulting in an increased morula/cleaved rate. Based on these findings, we hypothesize that, although HTF supported embryonic development in this species, the initial delay in cleavage may adversely affect the quality of these structures, being regarded as the least effective medium among the three tested.

The heterologous *in vitro* fertilization using TYH appears to have facilitated development with intermediate potential, as structures with more than eight cells were formed by Day 2, albeit in smaller quantities. Cleavage rates increased throughout the IVD. However, these structures did not achieve a high number of morulae by Day 5 due to an initial disadvantage. While the medium was likely stable for the species under study, it appears that some factors were still lacking in optimizing its performance as the most effective medium.

Finally, the TALP medium appears to have positively influenced the development of these zygotes, as evidenced by the high percentage of cleaved structures with more than eight cells observed by Day 2. Furthermore, by Day 5, structures with 2-7 cells had progressed to eight cells or morulae, and even the delayed structures underwent cleavage, resulting in a high percentage of cleaved structures over the 120 hours. Additionally, the number of morulae generated was comparable to the percentage of more developed structures observed on Day 2. Based on these findings, we can hypothesize that the TALP medium is the most stable for red-rumped agouti he-IVF, providing an environment that supports the subsequent development of the structures within the embryonic medium.

The lack of stabilization in the HTF and TYH media during he-IVF, caused by excessive glucose, may have led to a further disruption in the metabolic balance of these structures, thereby exacerbating oxidative stress, as reflected in our findings of ROS production and mitochondrial membrane potential. This effect was especially evident at the morula stage, where embryos fertilized in HTF (which has a higher glucose concentration) demonstrated increased ROS production and elevated mitochondrial membrane potential-both of which are recognized as detrimental to embryonic quality (Belli *et al*., 2019).

## CONCLUSION

In summary, TALP medium demonstrated superior performance compared to HTF and TYH during he-IVF in red-rumped agouti and guinea pig, enhancing embryonic development rates following IVD. Moreover, TALP was the only medium that provided a lower oxidative stress environment for the embryos throughout the 6-hour he-IVF. This study represents a significant advancement in understanding the reproductive dynamics of these species, providing substantial data that could support the future development of homologous IVF techniques for this wild rodent.
